# Association between location of prenatal care services and non-consented cesarean sections in Mexico: A secondary analysis of the National Survey on the Dynamics of Household Relationships 2016

**DOI:** 10.1371/journal.pone.0303052

**Published:** 2024-05-14

**Authors:** Marian Marian, Ramona L. Pérez

**Affiliations:** 1 Herbert Wertheim School of Public Health and Human Longevity Science, University of California San Diego, San Diego, CA, United States of America; 2 San Diego State University School of Public Health, San Diego, CA, United States of America; 3 Department of Anthropology, San Diego State University, San Diego, CA, United States of America; Universidade Federal de Sao Paulo, BRAZIL

## Abstract

**Background:**

Mexico has one of the world’s highest rates of cesarean section (C-section). Little is known about Mexico’s frequency of and risk factors for non-consented C-sections, a form of obstetric violence. We examined the prevalence of sociodemographic and obstetric-specific characteristics of Mexican women who delivered via C-section, as well as the association between the location of prenatal care services and experiencing a non-consented C-section.

**Methods:**

We conducted a secondary analysis of data collected from Mexico’s 2016 National Survey on the Dynamics of Household Relationships (ENDIREH 2016) of women who reported a C-section during their latest delivery. Adjusted logistic regressions were calculated to explore the associations between the location of prenatal care services and experiencing a non-consented cesarean delivery, stratifying by Indigenous belonging.

**Results:**

The sample size for this analysis was 10,256 ENDIREH respondents, with 9.1% not consenting to a C-section. ENDIREH respondents between the ages of 26 and 35 years old, living in urban settings, living in Central or Southern Mexico, and married or living with a partner experienced a higher prevalence of non-consented C-sections. For both women who identified as Indigenous and those who did not, the odds of experiencing a non-consented C-section were higher when receiving prenatal services in private settings. Receiving more than one type of prenatal service was also associated with increased odds of non-consented C-sections, while ENDIREH 2016 respondents who did not identify as Indigenous and received prenatal care at the State Institute for Social Security and Services for State Workers facility had lower odds of experiencing a non-consented C-section.

**Conclusions:**

This analysis indicates that receiving prenatal care at a private facility or a combination of public and private services increases the risk of experiencing a non-consented C-section in Mexico. Additional research is required to further understand the factors associated with non-consented C-sections in Mexico.

## Background

Every woman has the right to a pregnancy and childbirth process that is dignified and respectful. Evidence worldwide shows a persistent occurrence of obstetric violence in health care facilities [1,2]. Obstetric violence is the abuse, neglect, or mistreatment, including non-consented care, during the childbirth process [3,4]. This type of violence is a global issue, affecting women on all continents. Recent studies report a prevalence of various forms of obstetric violence that ranges between 18% and 75% in Brazil and Ethiopia, respectively, while in 12 European countries, on average, 12% of women suffered abuse and 24% expressed not being treated with dignity during delivery [5–7]. Obstetric violence could have immediate and long-term physical and psychological consequences in the life of the mother, such as pain and suffering from denial of pain relief medication or an episiotomy without anesthesia, sleeping problems, post-traumatic stress symptoms, and feelings of dehumanization that could result in distorted body perception and fear of future childbirths [8]. Non-consented care, which is the absence of an information process that enables a female patient to provide informed consent for a childbirth procedure, is one of the seven categories of disrespectful and abusive care during childbirth, a concept usually used interchangeably with obstetric violence [3].

The number of cesarean sections (C-sections) completed around the world continues to increase, with more than 20% of all births conducted through this surgical procedure [9]. In Egypt, Turkey, Brazil, the Dominican Republic, and Cyprus, the number of C-sections is higher than the number of vaginal deliveries.[10] In the Latin American and Caribbean region, C-sections make up more than 40% of the deliveries [10]. A study using the Robson classification, a system that classifies all deliveries into one of ten groups and compares C-section rates by type of facility and over time, two World Health Organization’s (WHO) multi-country surveys, found an increase in C-sections in 20 out of 21 countries from 2004 to 2010 [11,12]. It is expected that the number will increase to 29% by 2030, with the highest rates between 50% and 63% in Western and Eastern Asia, Latin America, and the Caribbean [9,10]. Globally, it has also been found that for-private health facilities perform more C-sections than non-profit ones, while women with private insurance also have higher rates of C-section deliveries compared to women with public health insurance [13,14]. While a C-section is a life-saving procedure when medically necessary, women and infants are at risk of short- and long-term health problems when C-sections are performed unnecessarily [9,15]. Since 1985, the WHO has provided a recommendation on the number of C-sections for every country [16]. This suggests the “ideal rate” for C-sections to be between 10% and 15%, as data shows that maternal and newborn deaths decrease when C-sections decrease towards 10% [16]. Women who are not informed or do not provide consent for their C-section suffer from the trauma of a surgical procedure that has long-term consequences for many when a non-consented cesarean section is performed on them.

The high number of C-sections is a growing problem in Mexico. Between 2008 and 2017, 45% of the deliveries in Mexico were completed through C-section, one of the highest rates in the world [17]. The number of unnecessary C-sections performed in public and private health care settings in Mexico has been increasing since 2002 [15]. Different individual-level factors in the Mexican population have been associated with C-sections, such as giving birth in a private setting, having early prenatal care services, and having high socioeconomic status and education [17]. Indigenous women in this country also experience higher rates of C-sections [17].

With rates between 18 and 33% [18,19], existing literature has shown a high prevalence of abuse and disrespect during childbirth delivery in Mexico; however, most of the literature has been qualitative or limited to a city or state [18–21]. Given the high rates of unnecessary C-sections in Mexico, identifying the occurrence and risk factors of non-consented C-sections on a larger scale could improve our understanding of the extent of the problem and how to address it. Prenatal care provides pregnant women with services such as screening, diagnosis, disease prevention, education, and counseling [22]. An outcome that may be modified by prenatal care is the method of birth delivery. This study used a nationally representative dataset to examine the association between the type of prenatal care received during pregnancy and receiving a non-consented C-section. Additionally, we conducted a stratified analysis by Indigenous belonging to better understand obstetric violence in this population, as literature shows that Indigenous women in Mexico are highly marginalized and reports of medical negligence and discrimination from the different health services to these women based on their gender and Indigenous belonging are well documented [23–26].

## Methods

### Study overview

This was a secondary analysis of the National Survey on the Dynamics of Household Relationships 2016 (ENDIREH 2016, by its initials in Spanish) data collection on obstetric violence. The study population consisted of ENDIREH 2016 respondents who delivered their last childbirth through a cesarean section in the last five years (2011–2016). We examined the association between the type of prenatal care received during pregnancy and experiencing a non-consented cesarean section during childbirth. This research builds on previous analyses [27,28] on ENDIREH 2016 and further informs on the situation of obstetric violence in Mexico in the form of non-consented cesarean sections.

### Study scenario

#### Country’s healthcare system

The healthcare system in Mexico is fragmented into three main components. The first one includes public services for the employed population through the Mexican Institute of Social Security (IMSS) and the Institute for Social Security and Services for State Workers (ISSSTE). IMSS provides medical coverage to people working in the formal private sector and their families [29], while ISSSTE provides services to the population working in the government sector as well as to their families [29]. The second component of Mexico’s healthcare system includes all the social programs from the government that provide services to the population without any type of insurance [29]. Community health centers financed by the federal government are examples of public healthcare facilities for people without insurance [30]. And third, the private sector. This includes service providers, such as physicians, clinics, and hospitals that charge for services [29]. Individuals who receive services from the private sector pay out of pocket or through private insurance, which may be offered by the employer or paid for by the individual.

### Dataset

ENDIREH 2016 was conducted by the Mexican National Institute of Statistics and Geography (INEGI, by its initials in Spanish) during the last trimester of 2016. This cross-sectional survey is representative at a national and state level [31] and studies various forms of violence against women in Mexico. ENDIREH 2016 collected information on experiences of physical, sexual, economic, patrimonial, and emotional violence, including obstetric violence. A probabilistic three-stage, stratified, and conglomerate sample design was used for ENDIREH 2016 [32]. More information on sampling design has been published on INEGI’s website [32]. The population interviewed were women aged 15 years and over and habitual residents of the selected housing unit in the sample who faced violence in different environments such as work, the community, or with their partner or family [33]. The survey measured the violence suffered by these women throughout their lives and the violence that occurred to them in the last 12 months. ENDIREH was previously completed in 2003, 2006, and 2011, but this is the first time that it included questions related to obstetric violence. ENDIREH 2016 was collected in person by trained female data collectors between October and November 2016 [33]. From the households selected for ENDIREH 2016, one female member per household was interviewed, with a response rate of 87.5% [33,34].

### Analytic sample

ENDIREH 2016 collected information on the experiences of physical, economic, sexual, patrimonial, and emotional violence among 111,256 women in Mexico. From these, 24,064 women between the ages of 15 and 49 gave birth to a child in the last five years (October 2011 to October 2016) and received obstetric care during their last childbirth. For this secondary analysis, women who delivered via vaginal birth during the last childbirth were excluded. ENDIREH 2016 respondents who stated not receiving any form of prenatal care during their last pregnancy were also excluded. The final sample size for this analysis was 10,256, which was the number of respondents who gave birth via cesarean section and received medical assistance during their last childbirth, which was 42.6% of ENDIREH 2016 respondents who gave birth with medical assistance in the last five years.

### Variables and outcomes

Independent variables included the sociodemographic and pregnancy and delivery variables of respondents. The sociodemographic variables included were age of the respondent at the time of the survey, Indigenous belonging, highest education level, marital status, PROSPERA status (Mexico’s conditional cash transfer program for people living in poverty), type of setting where respondents live, region where respondents live, and employment status at the time of the survey and during the past five years. INEGI developed three versions of the ENDIREH 2016: A) for women married or living in union; B) for separated, divorced, or widowed women; and C) for single women. For this analysis, marital status was determined by the type of instrument (A, B, or C) responded to. The independent variable, Indigenous belonging, which was captured by ENDIREH 2016 through the question “Based on your culture, do you consider yourself Indigenous?”, was collapsed for this analysis to be considered dichotomous (“yes/yes-partially” and “no/don’t know”). The independent variable of type of setting was also collapsed to be considered dichotomous (“urban/completely urban” and “rural”). The independent variable of region where respondents live was collapsed into four categories: North (Baja California, Baja California Sur, Coahuila, Chihuahua, Durango, Nuevo León, Sonora, Tamaulipas), South (Campeche, Hidalgo, Chiapas, Guerrero, Oaxaca, Puebla, Tlaxcala, Quintana Roo, Tabasco, Veracruz, Yucatán), Central (Aguascalientes, Colima, Guanajuato, Jalisco, Michoacán, Nayarit, Querétaro, Morelos, San Luis Potosí, Sinaloa y Zacatecas), and Ciudad de México and Estado de México. The 31 states that make up México were geographically collapsed into these categories, following the categorization used by Mexico’s INEGI and the National Institute of Public Health (INSP, its Spanish acronym) [35]. A socioeconomic status variable was not included in this analysis, as there was not a question on the ENDIREH 2016 that directly asked respondents about this sociodemographic aspect.

The variables related to pregnancy and delivery were the number of pregnancies between 2011 and 2016, the number of live births in the last five years, the number of miscarriages in the last five years, the number of stillbirths in the last five years, and place of last delivery.

Non-consented cesarean section was used as the outcome for the analyses. A cesarean section was considered non-consented if respondents answered “no” to at least one of two questions related to non-consented care included in ENDIREH 2016: “Were you informed in such a way for you to understand why a C-section was necessary?”; “Did you give permission or authorization for the C-section?”. Related to the variable on who provided authorization or permission for the C-section on the woman’s behalf, for ENDIREH respondents who did not provide authorization or permission for this procedure, the options were the ones present on the ENDIREH 2016 survey (“husband or partner”, “father, mother, or family member”, “another person”, and “no one”).

The location of prenatal care received was measured in two different ways. First, prenatal care was analyzed as a categorical variable using the options provided to the respondents in the survey (“community health center”, “IMSS”, “ISSSTE” clinic or hospital”, “state ISSSTE public hospital”, “medical clinic/dispensary”, “public clinic/hospital”, “private hospital/clinic/practice”, and “other”). “Physician at pharmacy” and “midwife/healer” were other options included in ENDIREH 2016 but were collapsed into “other” due to their low number of responses. An additional option was included for analysis, as the code “more than one” was used when respondents received prenatal care services from more than one type of setting. Additionally, we assessed prenatal care options, which were collapsed into four categories (“public”, “private”, “both”, and “other”). “Community health center”, “IMSS”, “ISSSTE”, “state ISSSTE public hospital”, and “other public clinic/hospital” were collapsed into the category of “public” since these are healthcare locations covered by Mexico’s public health system or are government-based. “Private hospital/clinic/practice” and “physician at pharmacy” were considered “private” prenatal care services because they require payment from the patient. For respondents who received both private and public prenatal services, the location of prenatal service was “both”. ENDIREH 2016 did not provide enough information to determine if the options provided in the survey, “midwife/healer” and “medical clinic/dispensary”, were considered private or public, so these two options were categorized as “other”.

### Statistical analyses

Descriptive statistics of sociodemographic and pregnancy-specific characteristics were shown as a combination of frequencies, percentages, standard deviation, and range. To measure the relationship between the independent variables and consent to C-section provided by ENDIREH 2016 respondents, a chi-square test was completed. A multicollinearity diagnostic test was used to assess for collinearity among independent variables. Bivariate (crude odds ratio [COR]) and multivariable (adjusted odds ratio [AOR]) logistic regression analyses were completed to assess the association between the location of prenatal care (independent variable) and experiencing a non-consented cesarean section (outcome). Three logistic regression models were run. Model I examined each of the options provided by ENDIREH 2016 for the location of prenatal care received. Model II looked at the independent variable in four broad categories (“public”, “private”, “both”, and “other”). Modell III examined all the options provided by ENDIREH 2016 for the independent variable, just as Model I, except for “medical clinic or dispensary” and “public clinic or hospital”, as these were not included as options for place of delivery in the survey; only ENDIREH 2016 respondents who stated the same type of setting used for prenatal and place of delivery were included in this final model. For the three models, a stratified analysis by Indigenous belonging was also performed. Results were reported as crude odds ratios and adjusted odds ratios with 95% confidence intervals. The p-value for the level of statistical significance was p<0.05. For the multivariate logistic regression analyses, all variables were entered simultaneously. In order to not overfit the model, only variables with a p-value <0.05 were chosen as covariables for the adjusted logistic regression analyses. Age of the respondent at the time of the survey, highest education level, type of setting where the respondent lives, region where respondent lives, marital status, PROSPERA status, employment at the time of the survey, employment in the past five years, live births between 2011 and 2016, and stillbirths between 2011 and 2016, were the variables controlled for. SPSS Version 28.0.1.0 was used for the statistical analyses.

### Ethical considerations

Ethical approval and consent to participate were not required since no primary data were collected for this research study. The secondary data used for this study is available in the public domain. More details regarding INEGI data and ethical standards are available on the agency’s website (http://en.www.inegi.org.mx).

## Results

### Sociodemographic and obstetric-specific characteristics of the sample population

Table 1 presents the sociodemographic and obstetric-specific characteristics of ENDIREH 2016 respondents who gave birth through a cesarean section during their last pregnancy in the last five years (2011 to 2016). More than 51% of the respondents were between the ages of 26 and 35 at the time of the survey. About 73% of the respondents did not consider themselves Indigenous or did not know if they were Indigenous. Related to education, 35% had completed middle school. In terms of the type of residency, 76% lived in an urban setting, and more than 70% lived in the central or southern region of Mexico. Most of the respondents were married or living with a partner (87%). Only 10% of the respondents were PROSPERA recipients. More than half of the respondents were not employed at the time of the survey (61%) or in the past five years (61%).

**Table 1:** Sociodemographic and obstetric-specific characteristics of respondents, National Survey on the Dynamics of Household Relationships (ENDIREH) 2016 (N = 10,256).

Sociodemographic characteristics	n (%)	Consent to cesarean section provided by respondent		p-value of χ2
		No n (%)	Yes n (%)	
**Age of respondent**				< 0.001
18 and under	275 (2.7)	55 (20.0)	220 (80.0)	
19–25	2683 (26.1)	463 (17.3)	2220 (82.7)	
26–35	5236 (51.2)	650 (12.4)	4586 (87.6)	
36–45	1999 (19.5)	222 (11.1)	1777 (88.9)	
46–49	63 (0.6)	12 (19.0)	51 (81.0)	
**Indigenous Belonging**				0.152
Yes/Yes, partially	2707 (26.4)	392 (14.5)	2315 (85.5)	
No/Don’t know	7549 (73.6)	1010 (13.4)	6539 (86.6)	
**Highest Education Level**				< 0.001
Up to 6^th^ grade	1434 (14.0)	238 (16.6)	1196 (83.4)	
Middle school	3633 (35.4)	530 (14.6)	3103 (85.4)	
High school	2475 (24.1)	336 (13.6)	2139 (86.4)	
Technical school	400 (3.9)	53 (13.3)	347 (86.7)	
Teacher training	154 (1.5)	12 (7.8)	142 (92.2)	
4-year college	1974 (19.2)	215 (10.9)	1759 (89.1)	
Graduate degree	186 (1.8)	18 (9.7)	168 (90.3)	
**Marital Status**				0.028
Single	464 (4.5)	64 (13.8)	400 (86.2)	
Married/Living with partner	8966 (87.4)	1200 (13.4)	7766 (86.6)	
Separated/Divorced/Widowed	826 (8.1)	138 (16.7)	688 (83.3)	
**Type of setting where respondent lives**				< 0.001
Rural	2415 (23.5)	384 (15.9)	2031 (84.1)	
Urban	7841 (76.5)	1018 (13.0)	6823 (87.0)	
**Region where respondent lives**				0.039
North	2418 (23.6)	324 (13.4)	2094 (86.6)	
Central	3714 (36.2)	468 (12.6)	3246 (87.4)	
Ciudad de México and Estado de México	476 (4.6)	67 (14.1)	409 (85.9)	
South	3648 (35.6)	543 (14.9)	3105 (85.1)	
**PROSPERA Status** ^**1**^				0.006
Yes	1074 (10.5)	176 (16.4)	898 (83.6)	
No	9182 (89.5)	1226 (13.4)	7956 (86.6)	
**Employment at time of the survey**				0.046
Yes	3949 (38.5)	506 (12.8)	3443 (87.2)	
No	6307 (61.5)	896 (14.2)	5411 (84.8)	
**Employment in the past five years**				< 0.001
Yes	6327 (61.7)	805 (12.7)	5522 (87.3)	
No	3929 (38.3)	597 (15.2)	3332 (84.8)	
**Obstetric characteristics**	**Mean**	**SD**	**Range**	**p-value of χ2**
**Number of pregnancies between 2011 and 2016**	1.27	0.52	1–6	0.114
**Live births between 2011 and 2016**	1.25	0.55	0–6	0.016
**Stillbirths between 2011 and 2016**	0.03	0.19	0–3	< 0.001
**Miscarriages between 2011 and 2016**	0.10	0.35	0–7	0.742
		**Consent to cesarean section provided by respondent**		
**Type of prenatal care provider**	**n (%)**	**No** **n (%)**	**Yes** **n (%)**	< 0.001
Community Health Center	2878 (28.1)	453 (15.7)	2425 (84.3)	
Mexican Institute of Social Security (IMSS) facility	2683 (26.2)	360 (13.4)	2323 (86.6)	
Institute for Social Security and Services for State Workers (ISSSTE) facility	292 (2.8)	30 (10.3)	262 (89.7)	
State Institute for Social Security and Services for State Workers (ISSSTE) facility	65 (0.6)	12 (18.5)	53 (81.5)	
Public Clinic or Hospital	1435 (14.0)	241(16.8)	1194 (83.2)	
Medical clinic or dispensary	107 (1.0)	15 (14.0)	92 (86.0)	
Private clinic, hospital, or medical office	2077 (20.3)	209 (10.1)	1868 (89.9)	
Midwife or healer	4 (0)	2 (50.0)	2 (50.0)	
Physician at pharmacy	23 (0.2)	4 (17.4)	19 (82.6)	
Other	145 (1.4)	18 (12.4)	127 (87.6)	
More than one	547 (5.3)	58 (10.6)	489 (89.4)	
**Place of last delivery**				< 0.001
Community Health Center	817 (8.0)	119 (14.6)	698 (85.4)	
Mexican Institute of Social Security (IMSS) facility	2851 (27.8)	404 (14.2)	2447 (85.8)	
Institute for Social Security and Services for State Workers (ISSSTE) facility	289 (2.8)	34 (11.8)	255 (88.2)	
State Institute for Social Security and Services for State Workers (ISSSTE) facility	94 (0.9)	16 (17.0)	78 (83.0)	
Other State Public Clinic or Hospital	3286 (32.0)	550 (16.7)	2736 (83.3)	
Private clinic, hospital, or medical office	2763 (26.9)	255 (9.2)	2508 (90.8)	
Other	156 (1.5)	24 (15.4)	132 (84.6)	
**Same type of location for prenatal care and place of delivery**				< 0.001
Yes	5432 (53.0)	629 (11.6)	4803 (88.4)	
No	4824 (47.0)	773 (16.0)	4051 (84.0)	

^**1**^PROSPERA was Mexico’s conditional cash transfer program for people living in poverty at the time of the interview (eliminated in 2019).

The mean for the number of pregnancies in our analytical sample was 1.27, and for live births, it was 1.25. About 72% of respondents received prenatal services exclusively at a public institution, while 20% only received prenatal services from a private facility. The three most common locations of prenatal care services received by respondents during their last pregnancy were community health centers (28%), followed by IMSS facilities (26%), and private facilities (20%). More than half of ENDIREH 2016 respondents (53%) gave birth in the same type of location as where they received prenatal care.

Related to the cesarean sections, 9.8% of respondents were not informed that a cesarean section was necessary, and 9.1% of the respondents did not give permission for a cesarean section. Among the women who did not give permission, in 61% of the cases, the husband or partner provided permission or authorization for a cesarean section. Table 2 presents these results.

**Table 2:** Prevalence of non-consented care during last childbirth among National Survey on the Dynamics of Household Relationships (ENDIREH) 2016 respondents (N = 10,256).

	n (%)
**Was not informed that cesarean section was necessary**	1001 (9.8)
**Respondent did not give permission for cesarean section**	932 (9.1)
**Person who provided permission or authorization for cesarean section on woman’s behalf (N = 947)**	
Husband/Partner	568 (60.9)
Father/Mother/Family member	160 (17.2)
Other	53 (5.7)
No one	151 (16.2)

### Factors associated with disrespect and abuse in the form of non-consented cesarean section

Compared to a community health center, receiving prenatal services at a private clinic, hospital, or medical office [AOR: 1.37; 95% CI: (1.13, 1.66)] was significantly associated with experiencing a non-consented cesarean section when controlling for age, highest education level, type of setting where the respondent lives, region where respondent lives, marital status, PROSPERA status, employment at the time of the survey, employment in the past five years, live births between 2011 and 2016, and stillbirths between 2011 and 2016 (Table 3). In the model stratified by Indigenous belonging, when controlling for the same variables, compared to a community health center, receiving prenatal services at a private clinic, hospital, or medical office was significantly associated with experiencing a non-consented cesarean section for people who identify as Indigenous [AOR: 1.56, 95% CI: (1.04, 2.33)] and people who are not of Indigenous belonging [AOR: 1.33, 95% CI: (1.07, 1.67)]. Compared to the reference, a community health center, the odds of experiencing a non-consented C-section were lower for women who did not identify as Indigenous and received prenatal services at a State ISSSTE facility [AOR: 0.46, 95% CI: (0.22, 0.94)] (Table 3).

**Table 3:** Bivariate and multivariate associations between location of prenatal care received and non-consented cesarean section during last childbirth within the Last 5 years among women who participated in National Survey on the Dynamics of Household Relationships (ENDIREH) 2016 (N = 10,256).

	Full dataset****				Stratified Analyses****							
					Indigenous Belonging							
					Yes				No			
**Location of prenatal care**	**COR**	**95% CI**	**AOR**	**95% CI**	**COR**	**95% CI**	**AOR**	**95% CI**	**COR**	**95% CI**	**AOR**	**95% CI**
Community health center (Reference)	1		1		1				1		1	
IMSS facility	1.20	(1.03–1.4)*	1.00	(0.85–1.18)	1.07	(0.81–1.40)	0.95	(0.70–1.28)	1.24	(1.03–1.48)*	1.01	(0.83–1.23)
ISSSTE facility	1.63	(1.10–2.41)*	1.21	(0.80–1.82)	3.50	(1.08–11.33)*	2.88	(0.85–9.70)	1.40	(0.92–2.14)	1.03	(0.66–1.60)
State ISSSTE facility	0.82	(0.43–1.55)	0.60	(0.31–1.16)	2.72	(0.35–20.86)	2.39	(0.27–20.72)	0.64	(0.32–1.27)	0.46	(0.22–0.94)*
Public clinic or hospital	0.92	(0.78–1.09)	0.89	(0.75–1.06)	1.10	(0.80–1.51)	1.01	(0.73–1.41)	0.86	(0.70–1.05)	0.84	(0.68–1.03)
Medical clinic or dispensary	1.14	(0.65–1.99)	1.15	(0.65–2.01)	1.05	(0.39–2.76)	0.97	(0.35–2.62)	1.18	(0.60–2.33)	1.16	(0.58–2.31)
Private clinic, hospital, or medical office	1.67	(1.40–1.98)***	1.37	(1.13–1.66)***	1.69	(1.17–2.44)**	1.56	(1.04–2.33)*	1.64	(1.34–2.01)***	1.33	(1.07–1.67)*
Other	1.15	(0.74–1.79)	1.04	(0.66–1.64)	1.47	(0.57–3.81)	1.42	(0.53–3.78)	1.05	(0.63–1.74)	0.95	(0.56–1.59)
More than one	1.57	(1.17–2.10)**	1.28	(0.94–1.73)	1.47	(0.79–2.75)	1.45	(0.75–2.80)	1.57	(1.13–2.19)**	1.25	(0.88–1.76)

* < 0.05

** < 0.01

*** < 0.001.

COR: Crude Odds Ratio; AOR: Adjusted Odds Ratio; CI: Confidence Interval.

****Controlling for age of the respondent, highest education level, type of setting where respondent lives, region where respondent lives, marital status, PROSPERA status, employment at time of the survey, employment in the past five years, live births between 2011 and 2016, and stillbirths between 2011 and 2016.

When looking at the collapsed groupings of prenatal care (private or public), receiving care at a private facility [AOR: 1.39; 95% CI: (1.18, 1.64)] or both public and private facilities [AOR: 1.56; 95% CI: (1.08, 2.27)] during prenatal care was associated with increased odds of experiencing a non-consented cesarean section, compared to only receiving prenatal care at a public clinic or hospital, after controlling for covariates (Table 4). For the stratified analyses controlling for the same variables, compared to a public prenatal care institution, receiving private services was significantly associated with experiencing a non-consented cesarean section for respondents regardless of Indigenous identity [Indigenous belonging: AOR: 1.48, 95% CI: (1.03, 2.13); non-Indigenous belonging: AOR: 1.39, 95% CI: (1.15, 1.67)]. Receiving both public and private services was also significantly associated with experiencing non-consented cesarean sections for respondents who are not of Indigenous belonging [AOR: 1.69, 95% CI: (1.10, 2.58)] when compared to a public institution (Table 4).

**Table 4:** Bivariate and multivariate association between location of prenatal services (private vs public vs both vs other vs no prenatal care received) and non-consented cesarean section during last childbirth within the last 5 years among women who participated in National Survey on the Dynamics of Household Relationships (ENDIREH) 2016 (N = 10,256).

	Full dataset****				Stratified Analyses****							
					Indigenous Belonging							
					Yes/Partially				No/Not sure			
**Location of prenatal care**	**COR**	**95% CI**	**AOR**	**95% CI**	**COR**	**95% CI**	**AOR**	**95% CI**	**COR**	**95% CI**	**AOR**	**95% CI**
Public (Reference)	1		1		1		1		1		1	
Private	1.55	(1.32–1.81)***	1.39	(1.18–1.64)***	1.55	(1.10–2.19)*	1.48	(1.03–2.13)*	1.54	(1.29–1.83)***	1.39	(1.15–1.67)***
Receive both types of services	1.87	(1.30–2.69)***	1.56	(1.08–2.27)*	1.35	(0.64–2.86)	1.22	(0.55- 2.68)	2.02	(1.33–3.07)***	1.69	(1.10–2.58)*
Other	1.10	(0.77–1.59)	1.13	(0.78–1.64)	1.10	(0.56–2.17)	1.08	(0.54- 2.19)	1.08	(0.72–1.70)	1.13	(0.73–1.74)

* < 0.05

** < 0.01

*** < 0.001.

COR: Crude Odds Ratio; AOR: Adjusted Odds Ratio; CI: Confidence Interval.

**** Controlling for age of respondent, highest education level, type of setting where respondent lives, region where respondent lives, marital status, PROSPERA status, employment at time of the survey, employment in the past five years, live births between 2011 and 2016, and stillbirths between 2011 and 2016.

For ENDIREH 2016 respondents who received prenatal care and gave birth at the same type of location, receiving prenatal care at a private clinic, hospital, or medical office [AOR: 1.45; 95% CI: (1.07, 1.97)] or more than one type of location [AOR: 2.01; 95% CI: (1.23, 3.29)] had significantly higher odds of experiencing a non-consented C-section compared to the community health center. When stratifying by Indigenous belonging, receiving prenatal care at a private clinic, hospital, or medical office was also associated with higher odds of experiencing a non-consented C-section among women who identify as Indigenous [AOR: 1.85; 95% CI: (1.02, 3.37)]. Women who did not identify as Indigenous had higher odds of experiencing non-consented C-sections compared to a community health center when receiving prenatal services at more than one type of location [AOR: 1.89; 95% CI: (1.06, 3.34)], while lower odds of experiencing this same form of obstetric violence when receiving prenatal services at a State ISSSTE facility [AOR: 0.31; 95% CI: (0.12, 0.78)] (Table 5).

**Table 5:** Bivariate and multivariate associations between location of prenatal care received and non-consented cesarean section during last childbirth within the last 5 years among women who participated in National Survey on the Dynamics of Household Relationships (ENDIREH) 2016 who received prenatal care and delivery services in the same type of setting (N = 5,432).

	Full dataset****				Stratified Analyses****							
					Indigenous Belonging							
					Yes				No			
**Location of prenatal care**	**COR**	**95% CI**	**AOR**	**95% CI**	**COR**	**95% CI**	**AOR**	**95% CI**	**COR**	**95% CI**	**AOR**	**95% CI**
Community health center (Reference)	1		1		1				1		1	
IMSS facility	0.99	(0.77–1.28)	0.84	(0.64–1.10)	1.03	(0.67–1.57)	0.89	(0.56–1.45)	0.95	(0.69–1.29)	0.79	(0.56–1.11)
ISSSTE facility	1.19	(0.74–1.90)	0.96	(0.58–1.58)	2.71	(0.79–9.23)	2.53	(0.69–9.40)	0.94	(0.55–1.60)	0.75	(0.42–1.33)
State ISSSTE facility	0.54	(0.25–1.18)	0.47	(0.21–1.07)	1.70	(0.21–13.87)	1.62	(0.15–18.04)	0.39	(0.16–0.92)*	0.31	(0.12–0.78)*
Private clinic, hospital, or medical office	1.66	(1.26–2.20)***	1.45	(1.07–1.97)*	1.92	(1.13–3.25)*	1.85	(1.02–3.37)*	1.52	(1.09–2.13)*	1.34	(0.92–1.93)
Other	0.89	(0.46–1.72)	0.80	(0.41–1.56)	0.99	(0.32–3.07)	0.78	(0.23–2.60)	0.84	(0.37–1.87)	0.73	(0.32–1.67)
More than one	2.39	(1.49–3.81)***	2.01	(1.23–3.29)**	2.46	(0.92–6.54)	2.69	(0.92–7.93)	2.24	(1.30–3.85)**	1.89	(1.06–3.34)*

* < 0.05

** < 0.01

*** < 0.001.

COR: Crude Odds Ratio; AOR: Adjusted Odds Ratio; CI: Confidence Interval.

****Controlling for age of the respondent, highest education level, type of setting where respondent lives, region where respondent lives, marital status, PROSPERA status, employment at time of the survey, employment in the past five years, live births between 2011 and 2016, and stillbirths between 2011 and 2016.

## Discussion

This study examined the relationship between non-consented cesarean sections and the location of prenatal care in Mexico using data from ENDIREH 2016, a large, nationally representative dataset. We found that almost 10% of respondents experienced a non-consented cesarean section. Further, we found that receipt of prenatal care at a private facility increased the odds of a non-consented cesarean section compared to care in a public facility. This relationship was also found when we stratified our sample by Indigenous belonging. Receiving prenatal services at a State ISSSTE facility was the only type of location with lower odds of experiencing non-consented C-sections for people who do not identify as Indigenous.

More than 1000 out of the 10,256 ENDIREH 2016 respondents who gave birth via a cesarean section were not informed that this procedure was necessary or did not provide permission for this surgical procedure. Our study supports the limited prior research showing the high burden of non-consented care globally. Research on disrespect and abuse during childbirth in Latin America, specifically in Peru, Brazil, and Venezuela, has found the lack of consent for all types of surgical procedures during childbirth, including non-consented C-sections, to be between 19% and 74% [36,37]. To the best of our knowledge, there is no literature that has focused exclusively on non-consented C-sections in other Latin American countries.

While the causes of non-consented care are not well understood, previous research has identified potential contributing factors. Higher age, higher socioeconomic level, and more years of schooling have been positively associated with planned C-section deliveries among Mexican women [38]. As described in the literature, the healthcare system in Mexico has a high number of patients with shortages in financial resources, supplies, medications, infrastructure, personnel, especially specialists, and poor distribution of services [39]. These, along with the lack of patience among healthcare providers, the lack of expertise in the use of vacuum extraction and forceps for assisted vaginal delivery, and trying to prevent medical liability, are reasons that have been linked as potential contributors to the high numbers of cesarean sections in both the public and private sectors [40] and could also be associated with the percentage of non-consented C-sections in Mexico found in the present analysis.

Our findings show that non-consented C-sections were more likely among ENDIREH 2016 respondents who received private prenatal care services. This finding is similar to a previous study that found that uninsured Mexican women had a higher risk of delivering via C-section in a private setting compared to giving birth in a public institution [40]. Factors related to the increased risk of non-consented C-sections in the private sector in Mexico could be related to the higher reimbursement rates of a cesarean section compared to vaginal delivery [15] and health insurance that only covers C-sections [15].

Our study also found that ENDIREH 2016 respondents who do not identify as Indigenous and received prenatal care services at a State ISSSTE facility during their last pregnancy had lower odds of experiencing non-consented C-sections. This was also found for respondents who received prenatal care and childbirth services in the same type of setting. According to INEGI data from 2020, 60% of the Mexican population received health care services from IMSS, ISSSTE, or state ISSSTE based on their work for the private, state, or federal sectors [41]. Previous research has found that women affiliated with ISSSTE or IMSS are more likely to have C-section deliveries, suggesting that giving birth either through a C-section or a vaginal delivery was affected by the institutional care policies; however, information specific to State ISSSTE is not known [40,42]. Research has also found that a considerable number of women with IMSS or ISSSTE insurance end up delivering in private settings [40]. Our findings show that only 53% of ENDIREH 2016 respondents gave birth in the same type of setting where they received prenatal services. Future ENDIREH surveys should include questions to identify the causes behind the change of setting and how it affects the informed consent process for planned and unplanned C-sections. Future studies should also examine in greater detail the risk of receiving a non-consented C-section based on the type of health insurance (or lack of it) as this could impact the decision on the location of prenatal care received, the institution where the delivery is completed, and enough information to understand why a C-section is necessary and to make an informed decision about it.

The results from our stratified analysis show the complexity behind the individual-level factors associated with C-sections and non-consented C-sections. The existing quantitative and qualitative research has shown the increased risk of C-sections for Indigenous women in Mexico [25,42,43]. This study shows that indeed, private prenatal service locations are associated with non-consented C-sections in women of Indigenous belonging. However, private prenatal location and a combination of public and private prenatal services were also found to be associated with forced C-sections in women who are not of Indigenous belonging. Also, while the rates of C-sections increased by more than 12% from 2011 to 2014 in locations with a higher presence of Indigenous populations, this has been found to be related to the introduction of universal health coverage in the country and an increase in C-sections and hospital births in poorer municipalities, where Indigenous women are more likely to live [17,44]. Still, for this study, when compared to public prenatal services, receiving prenatal services in a private location for women of Indigenous belonging was significantly associated with non-consented C-sections. While ENDIREH 2016 provided information on Indigenous belonging, for ethnic classification, more information is needed, such as the ethnic group the respondents are part of or, if applicable, the language spoken by the respondents who identify as Indigenous, to have a better understanding of how the different forms of obstetric violence, including non-consented C-section, are affecting the different ethnicities in the Mexican population. Further studies are required to better understand the individual-level and health system factors associated with non-consented cesarean sections.

Preventing obstetric violence is a topic of urgent matter on the Global Health agenda, as all women deserve to have a positive prenatal and childbirth experience with procedures that are explained and consented to. As the WHO explains, “interventions at the interpersonal level between a woman and a health care provider, as well as the level of the health care facility and health system” [45] are required for respectful maternity care that involves confidentiality, privacy, and dignity throughout the entire childbirth process. Findings from our research show an association between the type of prenatal care and non-consented C-sections in Mexico for women of Indigenous belonging and women who do not identify as Indigenous, a problem that can only be solved, as the WHO recommends, by working at all levels of the health care system.

The main limitation of this secondary analysis is the possible recall bias among respondents. This bias is likely to be higher among respondents who have given birth more than once, due to the possibility of combining past experiences. While it is important to state that the prevalence of non-consented cesarean sections among the respondents could be higher since ENDIREH 2016 only asked about the last pregnancy between 2011 and 2016, ignoring potential childbirth experiences. Due to the sensitive topics asked in ENDIREH 2016, there is also the possibility of the respondents being embarrassed or ashamed of the situation and not providing accurate information about it, increasing the potential for response bias. Another limitation to this study, as previously discussed in the literature, is the exclusion of ENDIREH 2016 respondents over the age of 45 who might have given birth in the past five years to answer the questions related to obstetric violence [27]. Not including a variable on socioeconomic status in the analysis, is also a limitation, as the literature has found an association between this variable and violence against women [46]. A final limitation to this analysis is the use of data collected in 2016, as more recent data from 2021 has been available; however, results from this analysis could be used to compare, guide, and inform future research done with 2021 data as well as future ENDIREH surveys. Despite the limitations, this secondary analysis had several strengths, including a large sample size that allowed for high statistical power. Another strength of ENDIREH 2016, as previously mentioned, was that this household survey was representative at a national level of all women aged 15 and older as well as of the population of each of the 32 states in the country [31]. To the authors’ knowledge, this is the first study that examined the potential association between the location of prenatal care received during pregnancy and experiencing a non-consented cesarean section during childbirth in Mexico, making an important contribution by using data from ENDIREH 2016.

## Conclusion

Our findings show that non-consented C-sections in Mexico were mostly associated with receiving prenatal care at a private facility or a combination of public and private services. It is essential to implement actions that improve the functioning of the different Mexican health systems at the prenatal and delivery service levels to ensure that every woman, regardless of the location where she receives her prenatal care, will have a respectful and non-abusive delivery with dignified care. Future ENDIREH surveys, along with the results of ENDIREH 2021, which was recently published and addresses changes in social services and health care systems that Mexico has gone through in the past five years, such as the elimination of PROSPERA and Seguro Popular (Mexico’s former governmental program for universal access to health services), will help build on the limited information about non-consented cesarean sections in this country and should further examine how consent and a decision for a C-section delivery were reached between the woman and the physician, as the number of unnecessary C-sections in Mexico continues to increase.
